# Pseudoangiomatous stromal hyperplasia presenting as a tumor

**DOI:** 10.1590/0100-3984.2017.0135

**Published:** 2019

**Authors:** Tatiane Cínthia Nascimento, Maria Célia Djahjah, Ana Helena P. C. Carneiro, Afrânio Coelho de Oliveira, Edson Marchiori

**Affiliations:** 1 Universidade Federal do Rio de Janeiro (UFRJ), Rio de Janeiro, RJ, Brazil.

Dear Editor,

A 12-year-old female patient presented to the breast disease department with a six-month history of a progressive, bilateral increase in breast volume. On physical examination, the breasts were seen to be large, pendulous, and asymmetrical, the right being larger than the left. Palpation revealed nodules that were mobile, with a fibroelastic consistency, in both breasts. Ultrasound showed multiple solid, circumscribed, bilateral nodules, one of which, located in the upper medial quadrant of the right breast, was submitted to core biopsy. The histopathological finding was juvenile fibroadenoma. Magnetic resonance imaging (MRI) identified multiple bilateral lobulated nodules with isointense signals in T1-weighted sequences; hyperintense signals in T2-weighted and STIR sequences; and moderate heterogeneous enhancement with a type I kinetic curve. The largest nodule was located in the upper inner quadrant of the right breast and measured 13 cm. The patient underwent bilateral breast nodule resection followed by breast reduction surgery. On microscopy, the lesions were seen to consist of numerous canaliculi with anastomoses, amid dense stromal collagen, covered by elongated myofibroblastic cells, without atypia or significant mitotic activity. Therefore, the diagnosis was pseudoangiomatous stromal hyperplasia presenting as a tumor. In the left breast, one of the lesions fit the pattern of a juvenile fibroadenoma, accompanied by foci of pseudoangiomatous stromal hyperplasia.

Pseudoangiomatous stromal hyperplasia of the breast is a benign proliferative lesion that can be an incidental finding in breast biopsies^(^^1^^)^. There have been few reports of pseudoangiomatous hyperplasia presenting as a tumor in young patients, the condition being more common in premenopausal or postmenopausal women who undergo hormone replacement, suggesting a relationship with hormonal factors^(^^2^^,^^3^^)^. It is typically unilateral and slow growing. In rare cases, it presents as diffuse masses, causing bilateral, asymmetrical breast enlargement^(^^2^^)^.

Recent studies in the radiology literature of Brazil have highlighted the importance of MRI in the evaluation of breast diseases^(^^4^^-^^7^^)^. The imaging findings of pseudoangiomatous hyperplasia are nonspecific^(^^8^^,^^9^^)^. On ultrasound, presents as a round or oval, circumscribed hypoechoic nodule. On mammography, it most commonly presents as a round or oval, circumscribed nodule, without calcification, or as focal asymmetry^(^^7^^-^^9^^)^. On MRI, the signal is variable in T1-weighted sequences and can be high in T2-weighted sequences. The post-contrast kinetic curve is usually type I^(^^3^^)^.

The differential diagnosis of pseudoangiomatous hyperplasia includes fibroadenoma (a benign fibroepithelial tumor), especially in young patients (because they present similar findings), and low-grade angiosarcoma, requiring histopathological correlation to confirm the diagnosis^(^^2^^,^^3^^,^^8^^)^.

In pseudoangiomatous hyperplasia, the fine needle aspiration findings are nonspecific and core biopsy has superior diagnostic accuracy^(^^10^^)^. However, in some cases, the appropriate histopathological diagnosis is made only after excision of the lesion^(^^11^^)^.

Pseudoangiomatous hyperplasia is characterized by dense proliferation of the breast stroma, forming anastomosing channels that can be mistaken for vascular spaces, without red blood cells, lined with cells that have no malignant characteristics^(^^2^^,^^3^^)^. Low-grade angiosarcoma presents vascular anastomosing channels containing blood cells, lined with atypical endothelial cells, and invading the adjacent mammary tissue^(^^1^^)^.

The management of pseudoangiomatous hyperplasia depends on its presentation. When it is an incidental finding, it can be monitored by ultrasound or mammography^(^^4^^)^. However, large lesions require excision. In cases of diffuse involvement of the breasts, mastectomy is recommended^(^^3^^,^^12^^)^.

**Figure 1:**
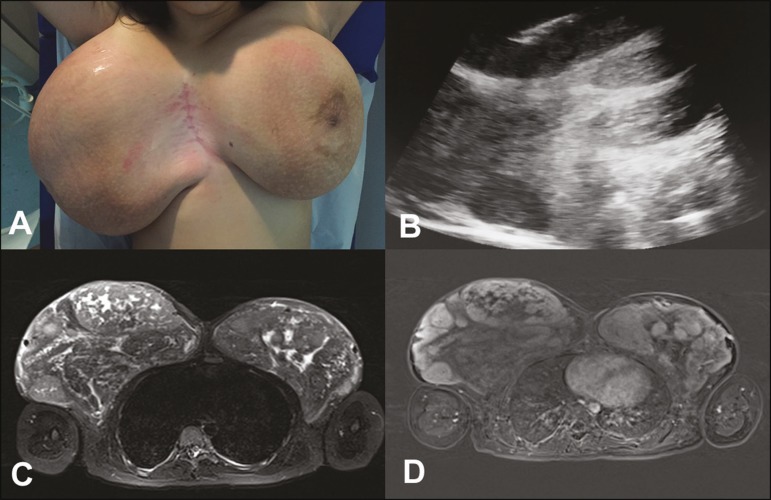
**A:** Photograph showing voluminous breasts, the right breast being larger than the left. **B:** Ultrasound of the right breast, showing solid nodules. Note the needle used in order to perform the core biopsy in the larger nodule. **C,D:** MRI of the breasts. Axial STIR sequence (**C**) showing multiple nodules in the breasts. Axial slice, with digital subtraction, acquired in the second minute of the dynamic study (**D**), showing heterogeneous enhancement of the nodules.
